# The Cow in the Room: Public Knowledge of the Links Between Dietary Choices and Health and Environmental Impacts

**DOI:** 10.4137/EHI.S914

**Published:** 2008-09-15

**Authors:** Andrew W Joyce, Sarah Dixon, Jude Comfort, Jonathan Hallett

**Affiliations:** 1 Department of Health Sciences, Monash University—Peninsula Campus; 2 School of Public Health, Curtin University of Technology, Western Australia

**Keywords:** climate change, public health, public knowledge

## Abstract

**Issue Addressed:**

This paper describes results of a survey comparing people’s knowledge of health and environmental impacts of dietary choices. Dietary choice is one of the key ways in which individuals can reduce their environmental impact in relation to water use and greenhouse gas emissions but this may not be widely known amongst the public due to limited press coverage.

**Methods:**

A street intercept survey was conducted asking open ended questions on how people can help the environment, maintain or improve health and basic demographics. The sample size was 107 with a refusal rate of 51%.

**Results:**

Only 3.2% of the sample made a link between dietary choice and environmental impact whereas 85.6% of the sample referred to dietary choice in relation to personal health. Transport options and keeping active were popular responses to both health and environmental categories.

**Conclusions:**

It seems that very few people are aware that the livestock sector is the second largest contributor to equivalent greenhouse gas emissions and one of the largest users of fresh water. Reduction in red meat consumption could have both important positive health and environmental impacts.

## Introduction

Surveys on public opinion on plant-based diets undertaken in Australia have shown that while the health benefits of plant-based foods may be recognised this may not be the case with the environmental benefits.^1^ While a reduction in meat consumption and increase in plant-based foods may offer protection against cardiovascular disease,^2^ type 2 diabetes^3^ and colorectal cancer,^4^ there are equally strong environmental reasons for increasing the consumption of plant-based foods and limiting meat consumption. The Food and Agricultural Organization of the United Nations produced an extensive review in 2006 of the impact of the livestock industry on environmental indicators.^5^ The livestock industry is a major contributor to global warming, emitting 18 percent of total greenhouse gas emissions, which is a higher share than transport.^5^ Climate change has been recognised as a substantial public health problem.^6^

The livestock sector is a major contributor to the water shortage, being responsible for over 8% of global water use.^5^ In Australia the dairy industry is the highest user of irrigated water in the Murray-Darling Basin^7^ and the livestock sector is a major contributor to water pollution, through animal wastes, antibiotics and hormones, fertilisers and pesticides used for feed cereals, and sediments from eroded pastures.^5^ Relative to producing vegetable based protein, animal based sources of protein require considerably greater use of water and land resources and produce greater wastes.^5^,^8^–^10^

McMichael et al. investigated ways to reduce the impact of livestock production on the environment.^11^ Improved environmental practices, such as improved practices in relation to reducing methane production from livestock enteric formation and manure management^12^ were cited as one recommendation, however current methane mitigation efficiency measures were noted as not producing the amount of change required to significantly impact on emissions.^11^ Thus it was proposed that developed countries significantly reduce their red meat consumption and that average consumption in developing countries aims to reach this lower target rather than matching current rates of consumption in developed countries.

Given the importance of dietary choice in respect to environmental and public health outcomes this study sought to examine people’s recognition of the link between diet choice, personal health, and environmental health. The study received Curtin University ethics approval (SPH-0017-2007).

## Method

A street intercept survey was conducted in Hay Street Mall, Perth, on Saturday 25th August 2007 between 11am and 3pm. The mall is located in the middle of the retail area of the CBD and it was expected that a broad cross-section of the community would be in this area of the city on the weekend. The survey was conducted by trained 3rd year health promotion students. The first two questions were open ended questions, ‘what can people do to help the environment on issues like climate change and water use?’ and ‘what can people do to improve or maintain good health?’ Respondents were read each question and the students were instructed not to provide any prompts. The other questions were closed questions and covered in order: age category, gender, occupation, interest in environmental issues, interest in health issues and personal diet. The items on interest in health and environmental issues were worded, ‘I am interested in environmental issues’ and ‘I am interested in looking after my own health.’ Responses were read out and scaled according to a likert scale ranging from strongly agree to strongly disagree. Participants were lastly asked, ‘how would you describe the way you eat, do you follow a particular diet?’ and read out the following options, ‘nothing special, weight loss, diabetic diet, vegan, vegetarian, try and eat healthy, and other.’

## Results

The sample size was 107 with a refusal rate of 51%. The gender breakdown was 46.2% females, 52.8% males and other 1%. The ages varied between 18 and 85 with over 50% of the sample between the ages of 18 and 35. There were 95.3% of respondents that strongly agreed or agreed that they were interested in health issues and 86.8% strongly agreed or agreed that they were interested in environmental issues. The responses for the diet question were try and eat healthy 50.5%, nothing special 33.3%, weight loss 5.7%, vegetarian 7.6%, diabetic diet 1.9% and other 1.0%. Table 1 presents information on respondents’ opinions on strategies to help the environment and improve or maintain good health. Individual responses were coded by the research team according to the broad categories presented in Table 1.

Table 1 outlines that while people associate dietary choice with health outcomes, very few made the same association with environmental impact. Transport options such as public transport, cycling, walking and car pooling were the most common responses by participants when asked how they could help the environment.

## Discussion

The livestock sector has a large impact on current environmental concerns of climate change and air pollution, water depletion and pollution, and biodiversity.^5^ One proposed action to combat climate change is that developed countries significantly reduce their red meat consumption and that developing countries aim to reach this lower target.^11^ At this stage it would appear that recognition of the impact of dietary choice is not readily made by the public.

Despite climate change having garnered public attention and livestock representing the second largest contributor per sectors, only four people from this sample made a link between dietary choice and environmental impact. This contrasted markedly with much higher knowledge on the importance of energy and transport use. This study was limited by the small sample size and as a street intercept survey was not systematic in recruiting a range of demographic characteristics among the sample. Further a forced choice option may have yielded a higher percentage of people making a diet and environment link. However, the very large discrepancy between knowledge of transport and energy compared to food choice suggests that information on this topic has been largely absent from public discourse with media coverage on climate change focusing on transport and energy sectors. Further research is required on assessing knowledge and attitudes around environmental impact of dietary options to inform communication messages. While education may not directly alter behaviour it may improve attitudes and knowledge and lead to increased support for economic, organisational and policy interventions that would be more effective in driving change.^13^

Any reduction in meat consumption made for environmental reasons may also improve public health profiles around chronic diseases of cardiovascular disease,^2^ type 2 diabetes^3^ and colorectal cancer.^4^ It may also result in a decreased threat of zoonotic disease.^11^ As McMichael et al. indicated such a campaign may also increase the percentage of people consuming vegetarian diets,^11^ which according to the American and Canadian Dietetic Associations is appropriate for all life-stages when well balanced.^14^

As a response to climate change and as a means of promoting health, urgent research is required on the communication messages that are likely to work in raising awareness of the importance of reducing red meat consumption in promoting both personal and environmental health. Such work presents a tremendous opportunity for health and environmental agencies to collaborate on campaigns to reduce the environmental impact of people’s dietary choices.

## Figures and Tables

**Table 1:** Percentage of respondents that listed at least one suggestion for maintaining health and helping the environment according to the following categories (n).

**What can people do to improve or maintain good health?**		**What can people do to help the environment such as climate change and water use?**	
Response	Percentage	Response	Percentage
Be active	87.5 (91)	Transport	59.6 (56)
***Diet related (healthy eating)***	***85.6*(*89*)**	***Diet***	***3.2*(*3*)**
Lose weight	5.8 (6)	Energy saving	46.8 (45)
Practice safe health behaviours	35.6 (37)	Water saving	58.5 (55)
Regular health checks	10.6 (11)	Waste/recycling	29.8 (28)
Look after mental health	14.4 (15)	Plant trees	13.8 (13)
Drink water	7.7 (8)	Consume less	38.3 (36)
Other	5.8 (6)	Other	16.0 (15)
